# Survival and predictors of death in tuberculosis/HIV coinfection cases in Porto Alegre, Brazil: A historical cohort from 2009 to 2013

**DOI:** 10.1371/journal.pgph.0000051

**Published:** 2021-11-10

**Authors:** Évelin Maria Brand, Maíra Rossetto, Bruna Hentges, Gerson Barreto Winkler, Erica Rosalba Mallmann Duarte, Lucas Cardoso da Silva, Andrea Fachel Leal, Daniela Riva Knauth, Danielle Lodi Silva, George Henrique Aliatti Mantese, Tiane Farias Volpato, Paulo Ricardo Bobek, Amanda Pereira Ferreira Dellanhese, Luciana Barcellos Teixeira

**Affiliations:** 1 Programa de Pós-Graduação em Saúde Coletiva, Universidade Federal do Rio Grande do Sul, Porto Alegre, Rio Grande do Sul, Brazil; 2 Department of Medicine, Universidade Federal da Fronteira Sul, Chapecó, Santa Catarina, Brazil; 3 Programa de Pós-Graduação em Epidemiologia, Universidade Federal do Rio Grande do Sul, Porto Alegre, Rio Grande do Sul, Brazil; 4 European Master in Health Economics and Management, Erasmus University Rotterdam, ERASMUS, Rotterdam, Netherlands; 5 Programa de Pós-Graduação em Políticas Públicas, Universidade Federal do Rio Grande do Sul, Porto Alegre, Rio Grande do Sul, Brazil; 6 Hospital Divina Providência, Porto Alegre, Rio Grande do Sul, Brazil; 7 Department of Public Health, Universidade Federal do Rio Grande do Sul, Porto Alegre, Rio Grande do Sul, Brazil; University of New Brunswick, CANADA

## Abstract

**Background:**

Tuberculosis is a curable disease, which remains the leading cause of death among infectious diseases worldwide, and it is the leading cause of death in people living with HIV. The purpose is to examine survival and predictors of death in Tuberculosis/HIV coinfection cases from 2009 to 2013.

**Methods:**

We estimated the survival of 2,417 TB/HIV coinfection cases in Porto Alegre, from diagnosis up to 85 months of follow-up. We estimated hazard ratios and survival curves.

**Results:**

The adjusted risk ratio (aRR) for death, by age, hospitalization, and Directly Observed Treatment was 4.58 for new cases (95% CI: 1.14–18.4), 4.51 for recurrence (95% CI: 1.11–18.4) and 4.53 for return after abandonment (95% CI: 1.12–18.4). The average survival time was 72.56 ± 1.57 months for those who underwent Directly Observed Treatment and 62.61 ± 0.77 for those who did not.

**Conclusions:**

Case classification, age, and hospitalization are predictors of death. The occurrence of Directly Observed Treatment was a protective factor that increased the probability of survival. Policies aimed at reducing the mortality of patients with TB/HIV coinfection are needed.

## Introduction

Tuberculosis (TB) is a curable disease, which remains the leading cause of death among infectious diseases worldwide, and it is the leading cause of death in people living with HIV (PLHIV) [1]. Although the global estimate was 10 million new TB cases for 2019, 7.1 million newly diagnosed people were reported, probably due to underreporting or underdiagnosis [2]. In the same year, the total number of deaths from TB was 1.4 million, of which 208,000 were due to PLHIV [3].

HIV infection is one of the main risk factors for TB [1, 2]. TB/HIV coinfection is a synergistic combination, in which one infection accelerates the progression of the other, bringing about a worsening of the clinical condition and possibly causing death, especially due to immunosuppression [4–6]. The reduction in incidence rates and tuberculosis mortality is one of the Millennium Development Goals [2, 7]. However, low- and middle-income countries face health inequalities, which make it difficult to meet these goals. The early detection of HIV cases and the timely initiation of antiretroviral therapy will help to reach this goal as they are fundamental actions to prevent the development of active tuberculosis and death [6, 8–12].

Since 2000, tuberculosis treatment has prevented more than 600 million deaths worldwide [3]. The WHO highlights that, in the Americas region, the incidence of TB is slowly escalating due to an increasing trend in Brazil, which is a country with a high level of TB ranking among the top 30 countries with the highest TB and HIV levels in the world [2]. Medicines for treating TB and HIV are available free of charge through the public health system [13, 14]. However, the issues surrounding TB and HIV treatments are complex and go beyond universal distribution [15–17]. The World Health Organization recommends that the percentage of those who drop out of treatment should not exceed 5% of new cases [3]. In 2018 this percentage was 11.6% in Brazil. Porto Alegre, in the south of Brazil, where this study was carried out, was the Brazilian city with the highest percentage of treatment abandonment (25.3%) [14]. Faced with this reality, Directly Observed Treatment (DOT) emerges as the main support and monitoring action for the treatment of people with TB, providing an opportunity to identify the risk of non-adherence.

In Brazil, 73,864 new TB cases were diagnosed in 2019, with an incidence coefficient of 35.0 cases/100,000 inhabitants, and a mortality coefficient of 2.2 cases/100,000 inhabitants [14]. Of the total diagnosed in 2019, 8.5% had TB/HIV coinfection. Porto Alegre is the Brazilian city with the fourth highest TB incidence coefficient (84.4 cases/100,000 inhabitants) and the third with the highest mortality rate due to the disease (5.3/100,000 inhabitants) [12]. Porto Alegre is also among the Brazilian state capitals with the highest AIDS detection rate. In 2019 the rate was 58.5 cases/100,000 inhabitants, 3.3 times higher than the national rate [18].

Death from TB is preventable, and studies on its incidence have pointed out the fragility of health services in providing care and recognizing social, economic, and treatment aspects associated with the disease [6, 9, 19–23]. The aim of this study is to investigate the probability of survival and the risk and protection factors in patients in Porto Alegre with TB/HIV coinfection, who were registered between 2009 and 2013. We bring new evidence to bear to help direct public policies to reduce mortality and inequalities, in line with The End TB Strategy, and the Millennium Development Goals for 2030 [3, 7, 24].

## Materials and methods

### Study design

This is a historical cohort study carried out with patients over 18 years of age diagnosed with TB/HIV coinfection in the city of Porto Alegre, from 2009 to 2013. The study ended in 2015, with a follow-up survival study of up to 85 months after the diagnosis. Considering that the notification of these diseases to the national surveillance system in Brazil is mandatory, the sample should correspond to the total number of coinfection cases in the city.

### Study population

The participants of this study are all patients with a diagnosis of TB/HIV coinfection in the city of Porto Alegre, aged over 18 years, and registered in the national health surveillance system from 2009 to 2013. The sample is expected to correspond to the totality of patients with coinfection, since TB and HIV are compulsory notification diseases in Brazil and, therefore, all patients diagnosed should be notified by the national surveillance system. Nevertheless, the existence of underreporting in this system should be considered, and so SINAN TB cases can be considered a proxy of TB cases in Brazil.

### Study procedures

The data were collected from public health system databases. The incidence of TB/HIV coinfection was obtained from the National Tuberculosis Notification System (SINAN-TB). In this same system, sociodemographic data, on the status upon entry into the surveillance system, and information on the incidence of other health problems were obtained. To discover data on the participants’ hospitalization over the study period, data were collected from the Hospital Information System (SIH). Information on TB/HIV mortality was obtained from the Brazilian National Mortality Information System (SIM). For greater reliability of the study data, information on the total number of coinfected patients and incidence of death was obtained from the National HIV/AIDS Notification System (SINAN-HIV/Aids). In order to link the databases, three personal information items were used: the patient’s full name, their mother’s name, and their registration number in the public health system [11, 25].

Patient follow-up occurred until the closure of the case in the tuberculosis surveillance system, which occurred after 12 months of follow-up, with the patient’s classification as cure, abandonment, death, multidrug-resistant TB, or transfer. The outcome of the study was death from HIV and/or TB. For more reliable information on TB/HIV mortality, all cases of coinfection were subsequently checked in the National Mortality Information System (SIM).

The sociodemographic and behavioral variables collected were: sex, age (at the time of diagnosis), education (measured in years of study), race/color, whether the patient was homeless, and whether they were alcohol abusers.

In relation to other diseases, information was collected whether the patients had any psychological disorders. Four classifications of entry into the health service were identified: new case, recurrence, return after abandonment, and transfer. Transfer cases consist of patients who had been transferred from another town or city to Porto Alegre. The variable “Indication of directly observed treatment (DOT)” included the indication, regardless of whether or not DOT had been performed. DOT performance information was also collected. Incidence of hospitalization was obtained for the entire period of the cohort.

### Statistical analyses

Statistical analyses were performed using SPSS software version 21.0 (IBM). Bivariate and multivariate Cox regression models were used to estimate the crude and adjusted Hazard Ratio values and to identify independent mortality predictors. Variables with p <0.20 in Cox bivariate regression analysis were considered for the Cox multiple regression analysis. The mathematical expression for the Cox model used was λ(t|X_i_) = λ_0_(t)exp(ß_1_X_i1_ + _•••_ + ß_p_X_ip_) = λ_0_(t)exp(X_i •_ ß). The p value of ≤5% was considered significant in the final model. The analysis and comparisons of survival time were performed using Kaplan Meier curves, in which individuals were followed up until the end of the period and were dichotomized into being censored (completing the follow-up) or an event (death) occurring. The probabilities of survival for the entire sample were estimated for up to 85 months. Statistical comparisons of the probability of surviving for up to 24 months were made for patients who did and did not undergo DOT, using the Log-Rank test, with a significance level of 5%.

### Ethics statement

Waiver was granted to apply the consent form to the participants, by the Research Ethics Committees. Our request considered that this is an extremely relevant topic for public health, and that the methodology is based on a retrospective study, with linkage of records in large health surveillance databases. The researchers respected all the ethical precepts of the research legislation in Brazil and the data were anonymized.

This study was approved by the Research Ethics Committee of the Federal University of Rio Grande do Sul (Opinion No. 952.907) and the Ethics Committee of the Municipality of Porto Alegre (Opinion No. 939.250).

## Results

Between 2009 and 2013, 2,417 cases of TB/HIV coinfection were reported in Porto Alegre. Of this total, 1,793 (74.2%) patients survived, and 624 (25.8%) died. In the crude analysis (Table 1), there is a positive association in the crude model between the outcome and age, low education level, entry status, performance and indication of DOT, and hospitalization.

**Table 1:** Sociodemographic and clinical characteristics associated with death in cases of TB/HIV coinfection, 2009–2013, Porto Alegre, Brazil.

Characteristics	Total	Survival	Died	p^a^	Crude Hazard Ratio	p^b^
	n = 2417	n = 1793 (74,2%)	n = 624 (25,8%)		(IC 95%)	
**Sex**				0,328		
Male	1586	1166(73,5)	420(26,5)		1,10 (0,93–1,29)	0,286
Female	831	627(75,5)	204(24,5)			
**Age**	38±9,91	37,21±9,52	39,63±10,51	<0,001	1,02 (1,01–1,03)	<0,001
**Years of schooling**				0,003		
Up to 7 years	1546	1129(73)	417(27)		2,45 (1,16–5,17)	0,019
8 to 11 years	629	490(77,9)	139(22,1)		1,97 (0,92–4,21)	0,081
12 years or more	59	52(88,1)	7(11,9)		1,00	
**Race/color**				0,598		
White	1356	1011(74,6)	345(25,4)		1,00	
Mixed	1053	776(73,7)	277(26,3)		1,04 (0,89–1,22)	0,650
**Mental illness**				0,907		
Yes	990	73(73,7)	26(26,3)		1,03 (0,69–1,53)	0,876
No	2312	1716(74,2)	596(25,8)		1,00	
**Entry Classification**				<0,001		
New Case	1389	978(70,4)	411(29,6)		5,94 (1,91–18,5)	0,002
Recurrence	351	253(72,1)	98(27,9)		5,40 (1,71–17,0)	0,004
Return after abandonment	620	508(81,9)	112(18,1)		3,46 (1,10–10,9)	0,034
Transfer	57	54(94,7)	3(5,3)		1,00	
**Alcohol abuse**				0,457		
Yes	620	453(73,1)	167(26,9)		1,07 (0,89–1,27)	0,479
No	1794	1338(74,6)	456(25,4)		1,00	
**DOT indicated**				<0,001		
Yes	626	513(81,9)	113(18,1)		0,60 (0,49–0,74)	<0,001
No	1784	1274(71,4)	510(28,6)		1,00	
**DOT performed**				<0,001		
Yes	405	341(84,2)	64(15,8)		0,56 (0,43–0,72)	<0,001
No	1993	1438(72,2)	555(27,8)		1,00	
**Hospitalization**				<0,001		
Yes	1258	768(61)	490(39)		3,78 (3,12–4,57)	<0,001
No	1159	1025(88,4)	134(11,6)		1,00	
**Number of hospitalization**	2(1–4)	2(1–4)	2(1–4)	0,904	0,98 (0,95–1,01)	0,111
**Length of hospitalization (days)**	36(16–80)	36(17–80)	35,5(16–77,3)	0,423	0,99 (0,99–1,00)	0,034

p^a^ value of Pearson’s chi-square test. p^b^ value of the crude analysis of the COX regression.

In the adjusted analysis (Table 2), schooling lost its statistical significance. Age at diagnosis, classification of entry, and hospitalization remained as risk factors and DOT as a protective factor. In terms of age at diagnosis, for each additional year there was an increase of 2% in the risk of death (AHR = 1.02; 95% CI = 1.01–1.03). New cases had 4.58 times risk of death (95% CI 1.14–18.4), recurrence had 4.51 times risk of death (95% CI = 1.11–18.4), return after abandonment had 4.54 risk of death (95% CI = 1.12–18.4), compared with transfer cases. The use of DOT presented 41% greater protection against death (95% CI = 0.45–0.77). The cases that were hospitalized during the follow-up had a 4.06 times greater risk of death than those that were not (95% CI = 3.28–5.04).

**Table 2:** Cox regression analysis for cases of TB/HIV coinfection, 2009–2013, Porto Alegre, Brazil.

	*Adjusted Hazard Ratio* (IC 95%)	P*
**Age**	1,02 (1,01–1,03)	<0,001
**Years os schooling**		
Up to 7 years	1,92 (0,91–4,07)	0,087
8 to 11 years	1,55 (0,72–3,32)	0,260
12 years or more	1,00	
**Entry Classification**		
New case	4,58 (1,14–18,4)	0,032
Recurrence	4,51 (1,11–18,4)	0,035
Return after abandonment	4,54 (1,12–18,4)	0,034
Transfer	1,00	
**DOT performed**		
Yes	0,59 (0,45–0,77)	<0,001
No	1,00	
**Hospitalization**		
Yes	4,06 (3,28–5,04)	<0,001
No	1,00	

* Significance level of 5%.

**Fig 1:**
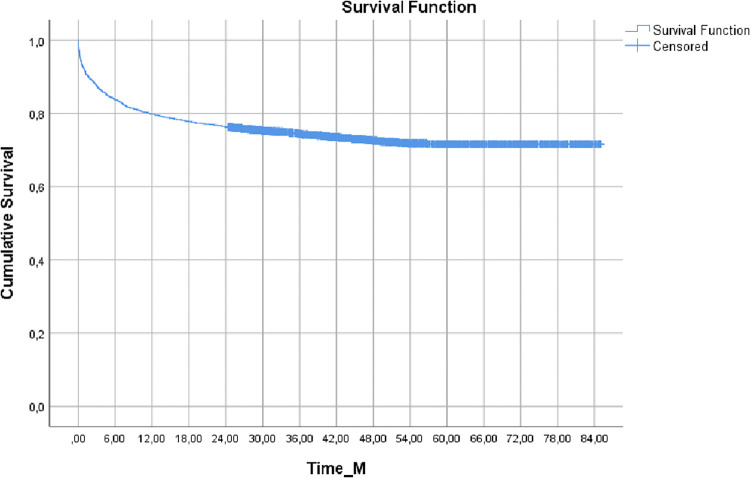
Cumulative survival of TB/HIV coinfection cases between 2009 and 2013, Porto Alegre, Brazil.

**Fig 2:**
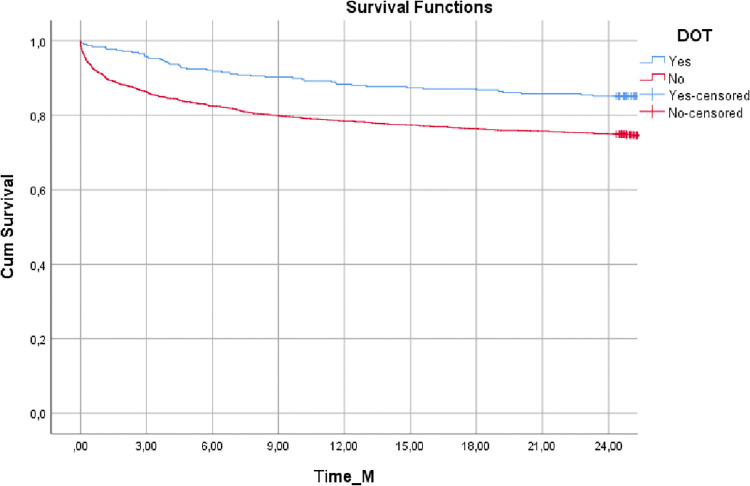
Survival curves adjusted according to the performance of DOT in cases of TB/HIV coinfection between 2009 and 2013, in Porto Alegre, Brazil. * p <0.001. Log-Rank test performed at 5% significance level.

Fig 1 shows the probability of surviving cumulatively over 85 months of follow-up. The median survival time was 63.94 ± 0.71 months. The 2,417 patients coinfected with TB/HIV had a 79.6% probability of survival over 12 months, and this was the period with the biggest drop in the probability of survival. At other times, the probability is similar: 76.7% at 24 months; 74.7% at 36 months; 72.7% at 48 months; and at other times stabilized at 71.9%. Fig 2 shows the probability of survival comparing those who performed DOT with those who did not over 24 months of follow-up, showing a significant difference between these two groups (p <0.001). The median survival time was 72.55 ± 1.57 months for patients who underwent DOT and 62.61 ± 0.77 for patients who did not.

Statistical difference in the probability of survival between patients who did or did not undergo DOT occurred at all times of the comparison (p <0.001). At 12 months, the probability was 86.9% for those who underwent DOT and 78.1% for those who did not. In 36 months, the probability was 84% for those who underwent DOT.

## Discussion

Our study showed a high mortality rate (25.8%) among patients coinfected with TB/HIV in the city of Porto Alegre. This finding corroborates data from Africa, the epicenter of the HIV-associated TB epidemic, where 20.2% mortality was observed in a study in Ethiopia [5], and also data from Asia, where 21% mortality was found in one study in Malaysia [26]. A higher incidence of mortality among coinfected persons was found in Myanmar (33%), which is one of the 30 countries with the highest rate of TB associated with HIV, but in patients who have not started ART [20].

The cumulative probability of surviving showed the greatest drop in the first 12 months of follow-up, at 79.6%. This probability falls over time, but remains above 70% right up to 84 months of follow-up. In a similar study conducted in Shanghai [27], the probability of survival of patients with TB/HIV coinfection was higher than in our study, 85% for the first 20 months. The decrease was greater in cases with delayed diagnosis of tuberculosis, indicating that access to diagnosis and treatment impacts survival.

Among the sociodemographic characteristics, the only one that remained associated with the outcome was age. For each additional year at the time of diagnosis, the risk of death increased by 2%. Other studies with similar results were found in Tokyo [28] and Ethiopia [5].

As for the classification of entry into the surveillance system, new cases had a higher risk of death, followed by cases of re-entry after abandonment, and those with recurrence of tuberculosis. This finding may show that the health services’ strategies for coping with tuberculosis are more focused on cases of greater vulnerability (which are those who have already been treated for tuberculosis and have been reinfected or those who have abandoned treatment) [3, 4]. However, a study conducted in Brazil between 2001 and 2011 found that patients with return after abandonment had a 1.69 times greater risk of death when compared to new cases [29]. Cases of recurrence and transfer did not show a significant association with mortality. However, it should be noted that our sample only included patients with coinfection, who present enormous challenges regarding adherence to treatment for TB and HIV [2, 3, 14].

Hospitalization is important for monitoring coinfected patients as it can show flaws in the outpatient care process. In the present study, the incidence of hospitalization represents a 4.06 times greater risk of death. This finding corroborates the research by Escada et al (2017), who observed an association between hospitalization and death in a cohort study conducted in Rio de Janeiro [21].

DOT has been a strategy recommended by the World Health Organization since 1993 as it promotes adherence to treatment mainly in cases of TB/HIV coinfection, and it has been shown to be a powerful strategy to prevent abandonment of treatment [6, 23, 30]. In Namibia, a country that, like Brazil, is also on the list of the 30 countries with the highest rate of TB and HIV, recent evidence has demonstrated the benefits of DOT [31, 32]. However, the WHO estimates that ART coverage for people with TB/HIV coinfection in Namibia is high, while in Brazil it is around 50% [3]. In Brazil, in order to reduce treatment dropout rates, the National Tuberculosis Control Program recommends DOT based on the patients’ vulnerability profile [13]. Considering that the present study showed that the use of DOT is a protection factor against mortality, the probability of survival was compared with patients who did not receive DOT.

A greater probability of survival was found in the group that underwent DOT. Although we have comparison data for the 84 months of follow-up, our emphasis in relation to DOT is in the first 12 months, when DOT is performed. We believe that patients could undergo longer treatment periods in the case of multidrug resistance, as well as in the cases where there is a recurrence of the disease or when treatment is abandoned, so it is important to monitor the likelihood of survival in the longer term. Thus, considering that the sample included only cases of TB/HIV coinfection, and assuming that adherence to TB treatment is similar [13], it seemed important to assess the entire follow-up period.

In Porto Alegre, in view of the decentralization of tuberculosis treatment, all new cases that were previously referred to a specialized service are now treated in primary care. Coinfection cases remain in the specialized services, but now it holds joint responsibility for care together with primary care. Decentralization of treatment favors adherence to treatment, the early detection of cases, assessment of contacts, prevention of adverse outcomes such as mortality and hospitalization, as well as the incidence of multidrug-resistant tuberculosis [13]. However, primary care seems to be experiencing poor performance in the treatment of this condition, due to factors such as the turnover of professionals, difficulty in user access to services, and poor diagnostic capacity [33].

Like all scientific research, our study has certain limitations. As this is a retrospective cohort study linked to secondary national databases, our results depended on the data available. Although the data used in this study are considered to be of high quality [3], we were unable to evaluate clinical information as this was not included in the databases used. Another possible limitation is that there was no detailed information in our databases regarding the arrangements between staff and patient in terms of DOT. For example, we do not know whether the actions were carried out all at the health units or whether home visit strategies were involved [13]. The literature shows new possibilities for carrying out DOT with electronic devices [34]. However, this is not yet the reality of our sample. Another important issue is that the DOT referred to tuberculosis. Since ART is universally and freely accessible in Brazil [18], it was considered that all patients would be receiving ART as the WHO recommends that ART begin within eight weeks for patients with TB/HIV. However, in this study it was not possible to add information about adherence to HIV treatment.

A suitable response to coinfection requires commitment and integration between specialized services that monitor TB and HIV and primary care, adequate screening in patients with HIV to exclude active TB, offering a rapid test for HIV diagnosis in patients with active TB, reliable supplies, and patient-centered support to ensure follow-up, adherence, and monitoring [35]. In Brazil, considerable effort is made to implement all these measures in order to achieve the HIV/AIDS and TB goals in the Millennium Development Goals agenda for 2030 [3, 7, 8]. In this sense, our study has added new evidence to contribute to public health policies.

## Conclusions

The mortality predictors identified for patients with TB/HIV coinfection were: the age of the person diagnosed, the classification of entry, and the incidence of hospitalization. DOT was identified as a protective factor against mortality, contributing to the probability of survival. This study therefore supports the adoption of DOT as a strategy to prevent mortality in all patients with TB/HIV coinfection. Considering resource limitations and the impossibility of carrying out DOT for all patients with TB/HIV coinfection, further investigations on death predictors are recommended in order to better define the priority groups that should be included in this care strategy.

## Supporting information

S1 Dataset Survival DOTS. (XLS)
